# Integrable Near-Infrared Photodetectors Based on Hybrid Erbium/Silicon Junctions

**DOI:** 10.3390/s18113755

**Published:** 2018-11-03

**Authors:** Mariano Gioffré, Giuseppe Coppola, Mario Iodice, Maurizio Casalino

**Affiliations:** Institute for Microelectronics and Microsystems, National Research Council, I-80131 Napoli, Italy; mariano.gioffre@cnr.it (M.G.); giuseppe.coppola@cnr.it (G.C.); mario.iodice@cnr.it (M.I.)

**Keywords:** silicon, near-infrared, photodetectors, internal photoemission, erbium

## Abstract

This paper presents the design, fabrication, and characterization of Schottky erbium/silicon photodetectors working at 1.55 µm. These erbium/silicon junctions are carefully characterized using both electric and optical measurements at room temperature. A Schottky barrier Φ_B_ of ~673 meV is extrapolated; the photodetectors show external responsivity of 0.55 mA/W at room temperature under an applied reverse bias of 8 V. In addition, the device performance is discussed in terms of normalized noise and noise-equivalent power. The proposed devices will pave the way towards the development of Er-based photodetectors and light sources to be monolithically integrated in the same silicon substrate, and both operating at 1.55 µm.

## 1. Introduction

Integrated photonics refers to the fabrication and integration of several photonic components on the same planar substrate. These components include beam splitters [1], gratings [2], couplers [3], interferometers [4], sources [5], and detectors [6], among others. In the last three decades, there has been growing interest in the use of silicon (Si) as a substrate for integrated photonics [7]. In this context, tremendous progress has been made in Complementary Metal Oxide Semiconductor (CMOS) technological processes based on the use of silicon-on- insulator (SOI) substrates. Now it’s possible to obtain reliable and effective optical components which are fully compatible with CMOS technology, such as low-loss waveguides, high-Q resonators, high-speed modulators, and couplers, typically operating at near-infrared wavelengths [5,8,9,10,11,12].

However, the silicon indirect band-gap and the minimal optical absorption at NIR wavelengths limit the use of Si for some active optical components like sources (leds, lasers) or NIR photodetectors (PDs).

The latter issue is typically addressed by the integration with Germanium (Ge). Unfortunately, the growth of Ge on a Si substrate is only achievable with a two-step epitaxial growth technique [13,14] that causes problems in both the thermal budget and planarity [6], hindering the monolithic integration of an electronic and photonic circuit on the same Si substrate. Indeed, Intel [15] and Luxtera [16] reported on a four-channel optical receiver working at 50 Gbps and 40 Gbps, respectively, based on Ge PDs that are separately fabricated, and only subsequently flip-chip-mounted to a Si electronic circuit. In addition, good progress has been made in fabricating SiGe-based PDs in such a “zero-change” CMOS process flow [17], with the Ge content of the available layer estimated to be 25% to 35% [18]; however, in this case, the wavelength of possible detection is limited to less than 1200 nm [19]. Thanks to this technological progress, a single-chip microprocessor integrating over 70 million transistors and 850 photonic components has been fabricated, taking advantage of SiGe PDs fabricated by a “zero-change” CMOS approach operating at 1180 nm [20]. Unfortunately, no Ge-based PDs operating at 1550 nm have been realized by a “zero change” approach as far.

In contrast to traditional semiconductors, lead sulfide (PbS) quantum dots (QD) combined with a conjugated polymer (MEH-PPV) have been used for infrared PDs [21]. These devices are characterized by a maximum responsivity of 3.1 × 10^−3^ A/W at 975 nm and under −5 V bias, showing also the capability of detecting longer wavelengths (980 nm, 1.200 μm and 1.355 μm) by varying the QD size. PbS QDs have also been deposited via a spin-casting process on top of a graphene layer in order to increase the sensitivity of a digital camera operating at both visible and short-wave wavelengths [22]. However, both PbS QDs and graphene have not yet fully demonstrated their automated integration in Si technology for volume fabrication. This is because, very often, an all-Si approach is preferred [23]. The internal photoemission effect (IPE) is commonly exploited as an option for the Si sub-bandgap detection [23]. IPE is the photo-excitation of the carriers in a metal layer to energies higher than the Schottky barrier and the emission of these carriers to the conduction band of the semiconductor [24]. In recent years, many IPE-based PDs on Si, including Schottky PDs [25], metal-semiconductor metal (MSM) PDs [26], and surface plasmon polariton (SPP) PDs [27], have been reported. However, their external responsivity rarely exceeds the tens of mA/W. Most recently, the use of graphene monolayers in place of metal has demonstrated the possibility of reaching responsivities as high as 0.37 A/W at 1550 nm [28]. It is worth mentioning that the aforementioned PDs are based on waveguide structures that are typically more responsive with respect to the vertically-illuminated counterpart, in which the light-matter interaction is strongly reduced [29]. 

On the other hand, thanks to the success of the so-called EDFA (Erbium-doped fiber amplifier) used in telecommunications, in recent years, much effort has been made to introduce Er in Si in order to realize Si-based light sources. Unfortunately, Si is not a good host for Er. The reason is believed to be linked to a back-transfer process of energy that strongly reduces the light emission at room temperature. The attractive features of this technology are the emission in the telecommunications band of 1550 nm, as well as electrical pumping. For this application, metal-oxide-semiconductor (MOS) structures are typically realized. A voltage applied between metal and Si causes electrons to tunnel through the oxide in which Er ions are dispersed, exciting them. In a subsequent version of the device, Si nanocrystals are dispersed together with Er ions in the oxide. Light emitting diodes based on this approach show efficiencies of about 10% as reported by STMicroelectronics [30], even if the light emission is observed to diminish when the current density increases. This effect is believed to be due to free carrier absorption and Auger processes; thus, despite preliminary successes, many concerns need still to be addressed.

Although Erbium is a very promising material for the realization of Si-based LASER, no investigations have been performed on the use of hybrid Er/Si structures in the field of near-infrared detection. Indeed, this could be very fascinating in the vision of integrating both Er-based LASERs and PDs on the same silicon substrate. In addition, Er can be deposited by sputtering at low temperature without compromising pre-existing electronic circuitry. On the other hand, sputtered Er deposited on Si has already been shown to be capable of forming Schottky junctions [31] that have been widely employed in both infrared and NIR detection [26,27,32,33,34].

In this work we report on Er-based Schottky PDs operating at 1550 nm and integrated with a double-polished 200 μm-thick Si substrate. Er has been deposited by a radio-frequency (RF) sputtering process at low temperature, and its optical properties have carefully been characterized by ellipsometric techniques. The photocurrent generation is based on the internal photoemission effect, where photoexcited carriers from Er are emitted into Si over the Schottky barrier Φ_B0_. The rectifying Schottky diode behavior is shown from the IV curve, from which both the series resistance and the ideality coefficients are extrapolated together with a Schottky barrier Φ_B0_ of ~673 meV. We show that an external responsivity (photogenerated current-incident optical power ratio) of R_ext_ ~0.29 mA/W at 1 V reverse bias can be obtained; this value increases to ~0.55 mA/W when a reverse voltage of 8 V is applied. We believe that this work paves the way for the realization of microsystems where Er-based sources and photodetectors are monolithically integrated onto the same Si substrate.

## 2. Materials and Methods

### 2.1. Erbium Deposition

The Erbium thin film was deposited directly on the Si substrate by a Radio-Frequency (RF) Sputtering technique from a 99.9% pure metal Er target, by an Argon gas process. The substrate was placed on the holder, and before the deposition process, the chamber was pumped down to a base pressure of 3 × 10^−6^ mbar. A 30 min presputtering process at 150 W RF power was necessary to overcome the target surface oxidation. An Er film was then deposited at room temperature, with 30 W RF power, at 2.5 × 10^−2^ mbar pressure, with a constant 40 sccm Ar flux and 11 min deposition time.

### 2.2. Erbium Ellipsometric Characterization

Spectroscopic ellipsometry data were recorded using a phase-modulated spectroscopic ellipsometer (UVSEL, Jobin Yvon Horiba, Palaiseau, France) with a wavelength scanning range from 300 to 1600 nm, at an incident angle of 70°. Acquired data were fit to an optical model of the sample in which the dispersion of the erbium film was assumed to follow a classical dispersion model [35,36], based on the sum of the single and double Lorentz, and Drude oscillators:

(1) $$\tilde{\varepsilon }\left(\omega \right)=\varepsilon_{\infty }+\frac{\left(\varepsilon_{s}-\varepsilon_{\infty }\right)\cdot \omega_{t}^{2}}{\omega_{t}^{2}-\omega^{2}+j\Gamma_{0}\cdot \omega }+\frac{\omega_{p}^{2}}{-\omega^{2}+j\Gamma_{d}\cdot \omega }+\overset{2}{\underset{i=1}{\sum }}\frac{f_{i}\cdot \omega_{0i}^{2}}{\omega_{0i}^{2}-\omega^{2}+j\cdot \gamma_{i}\cdot \omega }$$

The Lorentz oscillator model works well for insulators and semiconductors above the band gap, while the Drude model describes well the optical properties of metals but does not take into account the notion of optical band gap energy, *Eg*. The combination of both is often adequate when the material is a little conductive and has a metallic character. In our case, for the erbium dielectric function assessment, only one of the two oscillators of the last term was introduced. Fitting of experimental data leads to film thickness and dielectric function $\tilde{\varepsilon }=\varepsilon_{1}-j\varepsilon_{2}$ evaluation; the complex refractive index comes from the dielectric function, assuming that $\tilde{\varepsilon }=n^{2}={\left(n-jk\right)}^{2}$. The film structure used in the model consists of a homogeneous bulk erbium film and an outmost layer, described by a Bruggeman effective medium approximation (BEMA) [37].

### 2.3. Electrical Characterization

The PD electrical characterization consisted of the measurement of the current-voltage (I-V) characteristics of the Er/p-Si Schottky junction, where p-Si is biased with respect to grounded Er electrode. The I-V characteristics were acquired using a Keithley 6487 picoammeter. The I-V characteristic is the mean of five curves that were obtained by varying the voltages repetitively from −8 V to 2 V and vice-versa.

### 2.4. Optoelectronic Characterization

For optoelectronic characterization, a tunable NIR laser (AQ4321D, Ando, San Jose, CA, USA) was collimated, chopped, and split into two beams using a beam splitter. One beam was focused by a 20× IR microscope objective (MO) to the device under test (DUT). The transmitted light was collected using a 20× IR collecting MO and addressed on a NIR CCD to simplify the alignment procedure. A lock-in amplifier was used to measure the photocurrent produced by the DUT. The second beam was sent to a calibrated commercial power meter to perform incident optical power measurements, and consequently, to calculate the external responsivity.

## 3. Results and Discussion

### 3.1. Fabrication and Theoretical Background

**Figure 1:**
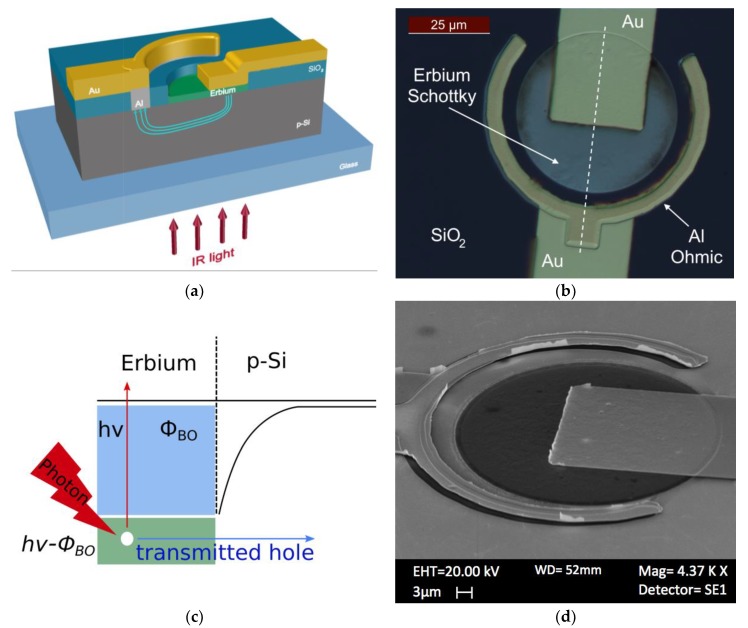
(**a**) Schematic cross-sectional view of the Erbium/Si Schottky PD under illumination; (**b**) optical image of a sample device; (**c**) the IPE mechanism in a Er/p-Si Schottky junction, where E_F_ is the Fermi energy of the metal, E_V_ is the Si valence band energy, and hν-Φ_B_ is the difference between the photon energy and the Schottky barrier; (**d**) SEM image of the device.

A bi-polished 200 μm-thick very lightly-doped P silicon was used to fabricate the device reported in Figure 1. A slightly doped substrate was chosen (~10^15^ cm^−3^) in order to avoid free carrier absorption.

The first step was a standard RCA cleaning process to remove any contaminants from the Si surface. Then, the Si substrate was thermally oxidized in order to obtain a 100 nm-thick silicon dioxide (SiO_2_). The two electrodes, the collecting Ohmic contact and the Schottky contact, were both realized on the top of the substrate. The collecting contact was made by a ring of 200 nm-thick aluminium film, thermally evaporated at 3 × 10^−6^ mbar and 150 °C.

First of all, the Shipley S1813 photoresist (PR) was deposited by spin coating at 4000 rpm, resulting in a PR thickness of 1.4 µm. Then, a standard photolithography process was used to form a ring pattern. After that, a SiO_2_ wet etching process was performed, and after the aluminium thermal evaporation, a lift-off process was carried out in order to obtain direct contact between Al and Si. Then, an annealing at 475 °C in nitrogen for 30 min, to obtain not-rectifying behaviour, was carried out.

The Schottky contact was fabricated by sputtering deposition of 50 nm-thick erbium film. The top of the wafer was covered by Shipley S1813 PR, exposed, and developed in order to obtain a disk surrounded by the Al ring Ohmic contact. Then, a SiO_2_ wet etching process was performed in order to deposit the Erbium in such a way that it was in direct contact with Si.

Then, the Erbium active materials were deposited as reported in the Section 2, and subsequently patterned. Finally, two gold (Au) electrodes have been realized by standard pholitography and lift-off process in order to connect both the Schottky (Er) and Ohmic (Al) contacts of the device to the macroscopic world. Finally, the sample was fixed onto a glass support, as shown in Figure 1a.

Internal quantum efficiency of Si-based Schottky PDs can be written as $\eta_{int}=C\cdot \frac{(hv-\Phi_{B)}{}^{2}}{hv}$ [38,39], where *C* is the quantum efficiency coefficient, *hν* is the energy photon, and Φ_B_ is the Schottky barrier height (SBH). On the other hand, *η_int_* is linked to the external responsivity $\eta_{ext}=A\cdot \eta_{int}$, where A is the metal absorption; this can be written as:

(2) $$A=\left(1-R\right)\cdot \left(1-e^{-\alpha \cdot d}\right)$$

where *R* is the reflectivity at the metal/Si interface, α and *d* are the absorption coefficient and thickness of the metal, respectively. The absorption coefficient α is linked to the penetration depth δ = 1/α α; thus, in the limit of metal thickness *d* >> δ, all optical power going into the metal will be absorbed and *A* = 1 − *R*. This means that the lower the reflectivity, the higher the absorption (and the higher the efficiency). The corresponding external responsivity can be written as [37]:

(3) $$R_{ext}=\frac{q}{hv}\cdot \eta_{ext}=\left(1-R\right)\cdot C\cdot \frac{{\left(hv-\Phi_{B}\right)}^{2}}{hv^{2}}\;\left[\frac{A}{W}\right]$$

### 3.2. Erbium Charactherization 

**Figure 2:**
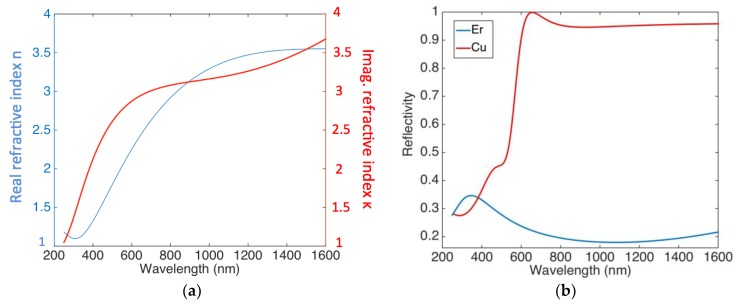
(**a**) Real (blue) and imaginary (red) refractive index of sputtered Er measured by ellipsometric characterization; (**b**) simulated reflectivity at both Si/Cu and Si/Er interface for normal incidence.

Figure 2 shows the ellipsometric characterization carried out on the sputtered Er in terms of the complex refractive index and reflectivity.

Figure 2a shows that Er is characterized by an extinction coefficient of κ = 3.68 at λ_0_ = 1550 nm; this leads to an absorption coefficient (α = 4πκ/λ_0_) and penetration depth (δ = 1/α) of 29.8 μm^−1^ and 33.6 nm at 1550 nm, respectively. By using the Fresnel coefficient for normal incidence, the reflectivity of the Si/Er interface is reported in Figure 2b, where the reflectivity of Si/Cu interface is also reported for comparison.

### 3.3. Electrical Charactherization 

**Figure 3:**
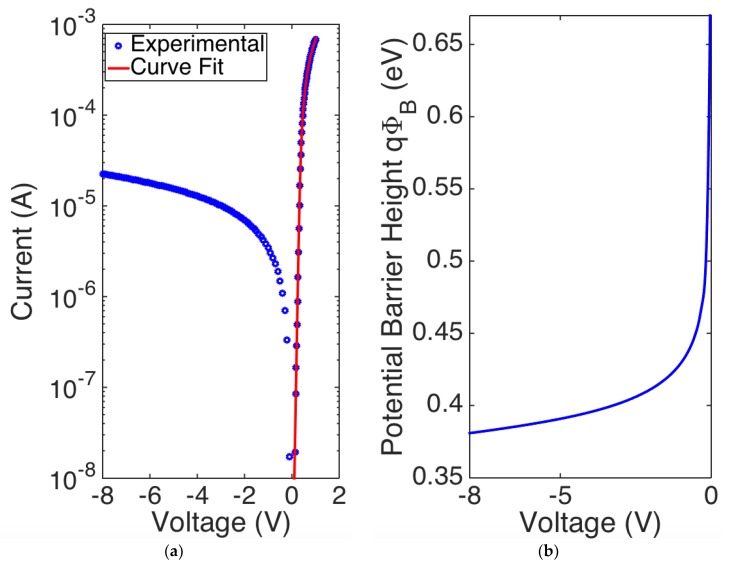
(**a**) I-V characteristics of Er/p-Si PD at room temperature; (**b**) potential barrier height as a function of reverse bias.

The Er/p-Si Schottky junction I-V experimental characteristics are shown in Figure 3a.

The device shows rectifying I-V diode behavior, which follows the Schottky diode equation [24]:

(4) $$I=I_{S}\cdot \left(e^{\frac{q\left(V-R_{S}I\right)}{\eta k_{B}T}}-1\right)$$

(5) $$I_{S}=AA^{*}T^{2}e^{-\frac{q\Phi_{B}}{k_{B}T}}$$

where $\Phi_{B}\left(V\right)=\Phi_{B0}-\Delta \Phi \left(V\right)$, $\Phi_{B0}$ is the SBH at zero voltage, ${\Delta \Phi }_{B}\left(V\right)$ is the SBH change due to applied voltage, *V* is the external applied voltage, *A* is the junction area, A* is the Richardson constant (32 A/cm^2^ K^2^ for p-type Si [40]), *η* is the diode ideality factor, defined as the deviation of the measured I-V curve from the ideal exponential behavior [40], and *k_B_T* ~ 26 meV at room temperature. In the low injection regime (*V* < 0.51 V), the device shows negligible series resistance, as highlighted from the linear behavior shown in Figure 3a; however, at higher voltages, the current deviates from the linearity, and *R_s_* cannot be neglected anymore. ΔΦ*_B(_V*) is significant in reverse bias where a barrier-lowering Schottky occurs due to image force effect [40], but it can be neglected in forward bias where Φ*_B_*~Φ*_B_*_0_ can be assumed. We estimate SBH in forward bias by fitting the experimental data with Equations (3) and (4), and by using Φ*_B_*, η and R_s_ as fitting parameters. We get Φ*_B_* = 0.673 ± 0.003 eV, *η* = 1.16 ± 0.01, and *R_s_* = 823 ± 3 Ω. An ideality factor η close to the unity indicates that the Er/p-Si junction has a behaviour that can be well approximated by the canonical equation of the Schottky junctions.

Figure 3b shows the potential barrier height as a function of reverse bias: it has been plotted by reverting Equation (5) and taking I_S_ as reverse current, which is experimentally measured and reported in Figure 3a. In the limit of *V*→0, this confirms a value of Φ*_B_*_0_ ~ 0.67 eV. Finally, Figure 3b shows the SBH dependence on the applied reverse voltage, and ΔΦ*_B_* up to ~0.29 eV at *V* = −8 V.

### 3.4. Electro-Optical Characterization

**Figure 4:**
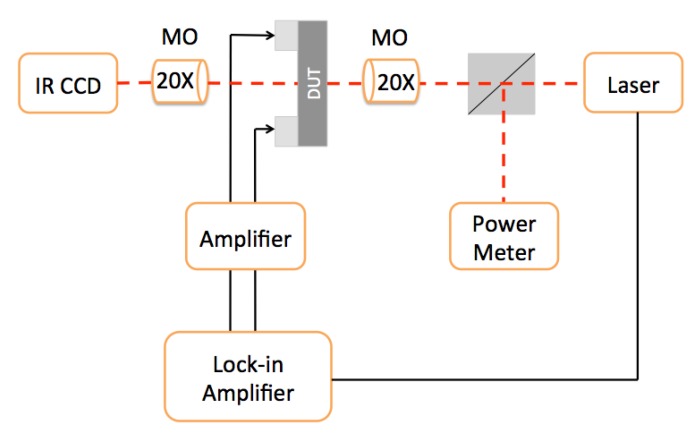
Experimental setup for opto-electronic experimental measurements.

**Figure 5:**
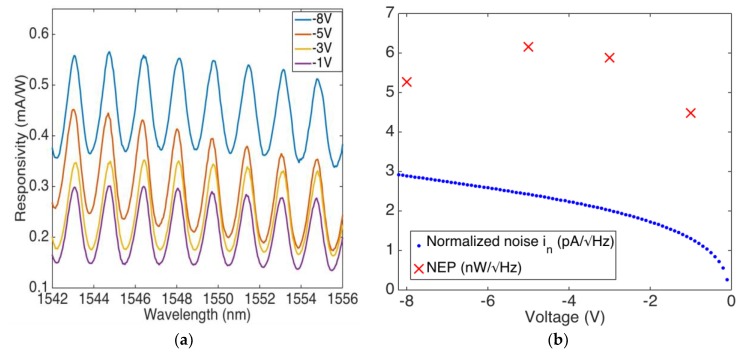
(**a**) Responsivity vs wavelength at different reverse biases; (**b**) NEP (red crosses) and total noise current in (blue dots) at different reverse biases.

The setup described in the Figure 4 and Section 2 was used for optoelectronic characterizations. Figure 5a plots the spectral response under various reverse voltages.

For any reverse voltage, the device shows many resonance fringes. These are due to the 200 µm-thick silicon layer behaving as a low finesse Fabry-Perot microcavity; thus, when the wavelength is in agreement with the cavity length, the optical field is enhanced in the Si cavity, leading to increased Er absorption. The spectral separation between the peaks, named Free Spectral Range (FSR), is ~1.7 nm in a very good agreement with the theoretical value of FSR = *λ*^2^/2*n_Si_L* = 1.73 nm, being *λ* = 1550 nm the wavelength, and *n_Si_* = 3.47 [39] and *L* = 200 μm the Si refractive index at 1550 nm and cavity length, respectively. As expected, at resonance we get photocurrent (responsivity) peaks due to increased absorption at the Er/Si interface. Maximum responsivity of around 1550 nm is ~0.3 at −1 V of the applied reverse bias.

To further enhance *R_ext_*, we exploit the Schottky barrier lowering effect and apply a larger (up to 8 V) reverse bias to the PDs.

Figure 5a shows *R_ext_* ~ 0.55 mA/W at *V* = −8 V around 1550 nm. It could be useful to mention that these devices show a responsivity more that is than two orders of magnitude higher than same device realized with copper (Cu) [29]. This remark can be partially explained by considering that the Er/Si interface is characterized by a reflectivity one order of magnitude lower than that of copper, as shown in Figure 2b. This leads to increased responsivity, as shown in Equation (3). It is worth noting that the slight change in peak height shown in Figure 5a is due to an overmodulation effect introduced by the glass support on which the sample is mounted, as shown in Figure 1a.

To estimate a noise figure of our PD, we calculate the amount of incident light power that generates a photocurrent equal to the noise current, i.e., the noise equivalent power (NEP), where NEP = *i_n_*/*R_ext_* [41]. Moreover, we assume that shot (quantum) and Johnson (thermal) noise dominates the low-frequency (1/*f*) noise [42,43]. The shot (*i_s_*) and Johnson (*i_j_*) noise currents normalized to the spectral band (1 Hz) are given by *i_s_* = (2*q*(*I_ph_* + I_d_))^1/2^ [24] and *i_j_* = ((4kT)/*R_eq_*)^1/2^ [24], where *I_ph_* is the photocurrent, *I_d_* is the dark current, *R_eq_* = *dV*/*dI* is the equivalent resistance of a PD at reverse bias in dark, and *i_n_ = i_j_* + *i_s_* is the normalized noise. Figure 5b plots both NEP and *i_n_* as a function of the reverse bias applied *V*, and for *V* = −8 V, we get *i_n_* ~ 2.9 pA/Hz^0.5^ and NEP ~ 5.3 nW/Hz^0.5^.

## 4. Conclusions

In conclusion, we have demonstrated a free-space Er-based PD for operation at 1.55 µm. The proposed devices are based on Er/pSi Schottky junctions, and their photodetection mechanism is based on internal photoemission. The Er/Si junction has been electrically characterized at room temperature, and a series resistance, ideality factor, and Schottky barrier of 823 Ω, 1.16 and 673 meV have been achieved, respectively. It is worth noting that the ideality factor is close to the unity demonstrating that the Er/pSi junction can be very well described, taking advantage of the canonical theory on the Schottky structures.

The PDs are characterized by external responsivity of 0.29 mA/W at 1 V; this value increases up to ~0.55 mA/W with a reverse voltage of 8 V. Finally, normalized noise and NEP have been discussed.

The results above indicate the possibility of the realization of Si-based free-space PDs that operate at 1.55 μm, taking advantage of the characteristics of the rare earth, Er. Moreover, it should be noted that the proposed device could be viewed as a low-finesse Fabry-Perot microcavity, where the Er absorption, and therefore, the device responsivity, can be periodically increased due to the enhancement of the optical field inside the Si cavity. In this context, responsivity may be enhanced through the use of high-finesse Fabry-Pérot optical microcavities, which have previously demonstrated their capabilities for the provision of greatly-increased absorption of the active medium [44]. In addition, the use of a high-finesse Fabry-Pérot optical microcavity would also be beneficial in terms of NEP.

The integration of Er in a standard CMOS process could be favored by the negligible thermal budget it requires, which has been deposited at room temperature. Since Er would be damaged during the high temperatures necessary for gate oxide growth, Er deposition should occur after gate processing but prior to the contact module. In this context and to achieve a full compatibility, it is mandatory to replace Au with metals that are compatible with CMOS technology. There are several advantages to monolithically integrating PDs with the rest of the receiver circuitry, including smaller parasitic capacitance and lower cost and scalability; the latter is very important for those applications where small-footprint PDs are required.

We believe that this work paves the way to the fabrication of Er-based sources and photodetectors that are monolithically integrated onto the same Si substrate.
